# Pin2 telomeric repeat factor 1-interacting telomerase inhibitor 1 (PinX1) inhibits nasopharyngeal cancer cell stemness: implication for cancer progression and therapeutic targeting

**DOI:** 10.1186/s13046-020-1530-3

**Published:** 2020-02-07

**Authors:** Chaosheng Yu, Fang Chen, Xiaoqi Wang, Zhimou Cai, Mengxue Yang, Qingwen Zhong, Jialian Feng, Junzheng Li, Congxiang Shen, Zhong Wen

**Affiliations:** 1 Department of Otorhinolaryngology-Head and Neck Surgery, Zhujiang Hospital, Southern Medical University, Guangzhou, 510282 China; 2 Department of Otorhinolaryngology-Head and Neck Surgery, Guangzhou Red Cross Hospital, Medical College, Jinan University, Guangzhou, 510235 China

**Keywords:** PinX1, Nasopharyngeal carcinoma, miR-200b, Migration, Invasion, Transcriptional regulation

## Abstract

**Background:**

Recurrence and distant metastasis are still the main factors leading to treatment failure for malignant tumors including nasopharyngeal carcinoma (NPC). Therefore, elucidating the molecular mechanisms underlying nasopharyngeal carcinoma metastasis is of great clinical significance for targeted gene therapy and prognostic evaluation. PinX1, a tumor suppressor gene, was previously demonstrated to be a powerful tool for targeting telomerase in order to resist malignant tumor proliferation and migration. The aim of this study was to explore the mechanism through which PinX1 regulates epithelial–mesenchymal transition (EMT) and tumor metastasis in NPC and investigate its clinical significance and biological role with respect to disease progression.

**Methods:**

Cell Counting Kit-8 (CCK8), Transwell assays, Colony formation analysis and Xenograft tumorigenicity assay were used to measure the nasopharyngeal CD133^+^ cancer stem cell proliferation, migration, and invasion abilities. Reverse transcription-quantitative polymerase chain reaction (RT-qPCR) and western blot assays were conducted to investigate the underlying mechanism that PinX1 inhibits cell proliferation, migration, and invasion via regulating EMT in nasopharyngeal CD133^+^ CSCs.

**Results:**

We found that the overexpression of PinX1 and P53 inhibited cell proliferation, migration, and invasion, but that the inhibition of miR-200b blocked these effects, in nasopharyngeal CD133^+^ cancer stem cells (CSCs). Mechanistic investigations elucidated that PinX1 inhibits cell proliferation, migration, and invasion by regulating the P53/miR-200b-mediated transcriptional suppression of Snail1, Twist1, and Zeb1, consequently inhibiting EMT in nasopharyngeal CD133^+^ CSCs.

**Conclusions:**

Our findings indicate that PinX1 inhibits cell proliferation, migration, and invasion via P53/miR-200b-regulated EMT in the malignant progression of human NPC, which might suggest novel clinical implications for disease treatment.

## Background

Nasopharyngeal carcinoma (NPC) is a malignant tumor of the head and neck, which is derived from epithelial cells located in the nasopharynx, accompanied by early distant metastasis and local invasion, and associated with a high incidence in southern China [1]. Some theoretical evidence demonstrates that the tumorigenesis of NPC is associated with Epstein-Barr virus infection, tumor suppressors, oncogenes, and environmental factors [2, 3]. Combined with chemotherapy technology, a comprehensive treatment plan based on intensity-modulated radiation therapy has achieved excellent local control of nasopharyngeal carcinoma [4]. However, tumor invasion and distant metastasis are still major challenges for successful treatment. Further, the molecular mechanisms underlying tumor invasion and metastasis in NPC need to be completely clarified.

Increasing evidence has indicated that the process of epithelial–mesenchymal transition (EMT), characterized by loss of the epithelial marker E-cadherin and the gain of mesenchymal markers vimentin and N-cadherin, plays an important role in the development of tumor invasion and metastasis for cells of various cancers including NPC [5–7]. Through EMT, epithelial cells lose cell polarity and adhesiveness and thus transform into mesenchymal cells, which might induce a cancer stem cell (CSC) phenotype in tumor cells [8], leading to invasive and metastatic CSCs [9, 10]. For example, Zhang et al. [11] demonstrated that leucine-rich repeat-containing G protein-coupled receptor 5 (LGR5), a stem cell marker for colon cancer and gastric cancer, can promote EMT by activating the Wnt/beta-catenin pathway in glioma stem cells. These findings suggest a vital link between EMT and the stemness of tumor cells [11, 12]. EMT and CSCs are key factors in cancer metastasis and invasion; however, the mechanism connecting EMT to stemness remains unclear. Therefore, it is imperative to investigate the molecular mechanisms that drive EMT and tumor-initiating capacity, which might have significant implications for the exploration of new therapeutic targets for the treatment of epithelial malignancies and metastasis.

In our previous work [13], we investigated the role of Pin2/telomeric repeat factor 1-interacting telomerase inhibitor 1 (PinX1) in nasopharyngeal CD133^+^ CSCs and found that its overexpression could inhibit proliferation, migration, and invasion and induce apoptosis by significantly downregulating c-Myc expression and upregulating TRF1, Mad1, and P53 expression. However, the underlying mechanisms through which PinX1 regulates EMT and stemness in NPC have not been fully elucidated.

MicroRNA-200b (miR-200b) has recently been found to be highly involved in EMT, tumor metastasis, CSC self-renewal, and differentiation [14–16]. For example, the overexpression of miR-200b was demonstrated to significantly inhibit tumor cell growth and differentiation by targeting GATA-4 to downregulate the expression of CCND1 [16]. In addition, miR-200b has also been found to suppress cell growth, migration, and invasion by targeting Notch1 in NPC [17]. Moreover, increased evidence indicates that the tumor-suppressor P53 can directly regulate miRNAs, which plays crucial roles in tumor initiation, progression, and metastasis [18, 19]. Accordingly, we hypothesized that PinX1 might regulate EMT and tumor metastasis in NPC through the functions of miR-200b and P53.

Currently, few studies have explored the potential mechanisms associated with the cooperation among PinX1, miR-200b, and P53 during the regulation of EMT and tumor metastasis in NPC. Therefore, we investigated the effects of PinX1, miR-200b, and P53 on EMT in nasopharyngeal CD133^+^ CSCs, aiming to provide new therapeutic targets to prevent distant metastasis and NPC progression. In this study, we found that the overexpression of PinX1 and P53 could inhibit nasopharyngeal CD133^+^ CSC proliferation, migration, and invasion but that the inhibition of miR-200b blocked these effects. Furthermore, we showed that PinX1 inhibits cell proliferation, migration, and invasion by regulating the P53/miR-200b-mediated transcriptional suppression of Snail1, Twist11, and Zeb1, ultimately inhibiting EMT to repress the migration and invasion of these cells.

## Materials and methods

### Cell culture and transfection

Nasopharyngeal CD133^+^ cancer stem cells (CSCs) and CD133^−^ CSCs were sorted from the nasopharyngeal cancer cell line CNE2 (poorly differentiated nasopharyngeal squamous cell carcinoma cell line; Beijing Concord Cell Resource Center) using magnetic beads in our previous work [13]. The cells were cultured in DMEM (HyClone, USA) supplemented with 5% fetal calf serum (Gibco, USA) in a humidified incubator with 5% CO_2_ at 37 °C. The culture medium was replaced after 24 h, and cells were passaged after 72 h. Third passages of nasopharyngeal CSCs at the logarithmic phase of growth were divided into the following groups: CD133^−^ CSCs (CD133^−^ CSCs without any transfection), blank (CD133^+^ CSCs without any transfection), negative control (NC, CD133^+^ CSCs transfected with empty vector), PinX1 overexpression (CD133^+^ CSCs transfected with pcDNA3.0-PinX1), P53 overexpression CD133^+^ CSCs transfected with pcDNA3.0-P53), miR-200b inhibitor (CD133^+^ CSCs transfected with miR-200b inhibitor), PinX1 overexpression + P53 overexpression (CD133^+^ CSCs transfected with pcDNA3.0-PinX1 and pcDNA3.0-P53) and PinX1 overexpression + miR-200b inhibitor (CD133^+^ CSCs transfected with pcDNA3.0-PinX1 and miR-200 inhibitor). The plasmids used were synthetized by Shanghai Sangon Biological Engineering Technology & Services Co., Ltd. (Shanghai, China). All plasmids were transfected into cells according to the instructions of Lipofectamine™ 2000 (Invitrogen, Carlsbad, California, USA). After transfection for 48 h, the cells were harvested and used for subsequent studies. The miR-200b inhibitor (5′-AGAGCUAGCACCAGUAUUA-3′) was designed and synthesized by Suzhou GenePharma Co.,Ltd. (Suzhou, China).

### CCK8 analysis

The proliferative capacity of nasopharyngeal CD133^+^ CSCs and the transfected CSCs was measured by Cell Counting Kit-8 (CCK8, GlpBio, USA) analysis. Briefly, after culture for 24, 48, and 72 h, the cells were seeded on 96-well plates at 1 × 10^4^ cells per well. CCK8 was added to the plates and the plates were incubated for 2 h. The absorbance at 450 nm was determined using a microplate reader. The experiment was repeated three times to obtain the mean values. The time point was regarded as the abscissa, the OD value as the ordinate, and cell viability curves were plotted.

### Migration and invasion assays

The migration and invasion of nasopharyngeal CD133^+^ CSCs and transfected CSCs were determined by Transwell assays. For migration analysis, Six hundred microliters of DMEM supplemented with 10% fetal bovine serum (FBS) was added to the lower chamber, while 2 × 10^4^ cells in serum-free medium were added to the upper chamber. The protocol for the invasion analysis was similar to that of migration analysis, except that the chambers were covered with Matrigel matrix (BD Biosciences, USA). During incubation, the cells migrated and invaded through the lower membrane. Cells on the lower chambers were stained and fixed with 4% paraformaldehyde and 0.1% crystal violet, which was followed by counting under an OLYMPUS CX41 upright microscope. A minimum of four fields of vision were randomly selected from each sample to calculate the mean number of cells that had moved through the Matrigel as the index of cell invasion ability.

### Colony formation analysis

Nasopharyngeal CD133^+^ CSCs and transfected CSCs were plated in 6-well plates at 1 × 10^4^ cells per well. The cells were incubated with serum-free DMEM/F12 medium supplemented with HGF (20 ng/mL), bFGF (20 ng/mL), insulin (10 ng/mL), and B27 and cultured for 10 days in a 5% CO_2_ humidified incubator at 37 °C. After colony formation was observed, the medium was removed. Cells were washed twice with PBS, fixed with 4% formaldehyde, and stained with 5% crystal violet. Colonies containing > 50 cells were used for counting.

### Xenograft tumorigenicity assay in nude mice

Four-week-old female nude mice with body weight of 17 g were randomly numbered using earrings. A total amount of 1 × 10^4^ logarithmically growing CD133^+^ CSCs without any transfection or transfected with pcDNA3.0-PinX1 and transfected with pcDNA3.0-PinX1 and miR-200 inhibitor in 0.1 ml 1640 medium without FBS were subcutaneously injected into the right side of the same nude mice (*N* = 5 each group), respectively, and tumor size was measured once a week in the feeding environment. At 4 weeks after injection, nude mice were sacrificed and tumor grafts were isolated. The size of tumor grafts was measured using the eq. V = (a^2^*b)/2, where a is the short side length of the tumor graft and b is long side length of the tumor graft. Differences in volume of tumor graft in CD133^+^ CSCs and CD133^+^ CSCs transfected with pcDNA3.0-PinX1 and CD133^+^ CSCs transfected with pcDNA3.0-PinX1 and miR-200b inhibitor injected sides were compared. The animals were provided by the Animal Laboratory of the Southern Medical University. In vivo experiments were approved by the Laboratory Animal Committee and conducted in accordance with the National Laboratory Animal Care and Maintenance Guide.

### Reverse transcription-quantitative polymerase chain reaction (RT-qPCR)

Total RNA was extracted from cultured cells with TRIzol reagent (Invitrogen) according to the manufacturer’s instructions and was used as a template for reverse transcription reactions into cDNA following the instructions of the Bestar qPCR RT Kit (Applied Biosystems, Grand Island, NY, USA). RT-qPCR was performed using the Agilent Stratagene Mx3000 real-time qPCR Thermocycle Instrument (Agilent Stratagene, CA, USA) with cDNA as the template and glyceraldehyde-3-phosphate dehydrogenase (*GAPDH*) as the internal reference. The PCR amplifications were performed using DBI Bestar® SybrGreen qPCRmasterMix. The reaction conditions were as follows: pre-degeneration at 95 °C for 2 min, and 40 cycles of denaturation at 94 °C for 30 s, annealing at 58 °C for 20 s, and extension at 72 °C for 20 s, followed by a final extension at 72 °C for 10 min. The average threshold cycle (Ct) values from three PCR assays were determined and results were calculated based on the 2^−ΔΔCt^ method and normalized to *GAPDH* levels. Primers for *PinX1*, *E-cadherin*, *Vimentin*, *Snail1*, *Twist1*, *Zeb1*, and *GAPDH* were designed and synthesized by Shanghai Sangon Biotechnology Co., Ltd. (Shanghai, China) (Table 1).

**Table 1:** The primer sequences for relative mRNA used in this study

ID	Sequence (5′-3′)	Product Length(bp)
*PinX1* F	CCAGAGGAGAACGAAACCACG	128
*PinX1* R	ACCTGCGTCTCAGAAATGTCA	
*E-cadherin* F	ACAGGGAGGATTTTGAGCAC	107
*E-cadherin* R	GATCAGCAGAAGTGTCCCTG	
*Vimentin* F	AGTCCACTGAGTACCGGAGAC	98
*Vimentin* R	CATTTCACGCATCTGGCGTTC	
*Snail1* F	ACTGCAACAAGGAATACCTCAG	242
*Snail1* R	GCACTGGTACTTCTTGACATCTG	
*Twist1* F	GAGCAAGATTCAGACCCTCA	115
*Twist1* R	CTCGTGAGCCACATAGCTG	
*Zeb1* F	CAGCTTGATACCTGTGAATGGG	106
*Zeb1* R	TATCTGTGGTCGTGTGGGACT	
*GAPDH* F	TGTTCGTCATGGGTGTGAAC	154
*GAPDH* R	ATGGCATGGACTGTGGTCAT	

### Western blot analysis

Total protein was extracted from 1 × 10^6^ cells using the Radio-Immunoprecipitation Assay (RIPA) lysate (Beyotime, Nanjing, China). Next, protein concentration was determined using a BCA Protein Assay Kit (Beyotime). The pre-treated proteins were added to the sampling wells (each well approximately 20 μg) for protein isolation on a 10% separation gel (120 V) and 5% spacer gel (100 V) for approximately 2 h. The protein samples were then transferred onto polyvinylidene fluoride membranes (Millipore, USA) and blocked with 5% non-fat milk for 1.5 h. Next, the membranes were washed and incubated with primary antibodies including rabbit polyclonal anti-PinX1 (dilution, 1:1000), rabbit monoclonal anti-Zeb1 (dilution, 1:1000), rabbit monoclonal anti-Snail1 (dilution, 1:500), rabbit monoclonal anti-E-cadherin (dilution, 1:3000), rabbit monoclonal anti-Vimentin (dilution, 1: 1500), rabbit polyclonal anti-Twist1 (dilution, 1:2000) and rabbit monoclonal anti-GAPDH (dilution, 1:10000) at 4 °C overnight. The membranes were then washed and incubated with the horseradish peroxidase (HRP)-labeled goat anti-rabbit immunoglobulin G (IgG) secondary antibody (dilution, 1:20000, ab6721) at 37 °C for 4 h. All aforementioned antibodies were purchased from Abcam Inc. (Cambridge, MA, USA). The target signals were visualized using an enhanced chemiluminescence detection kit (ECL, Beyotime). Densitometric analysis of the bands was carried out using the Gel imaging analysis system. Next, the Gel Doc XR imager system (Bio-Rad Laboratories, Inc., Hercules, CA, USA) was used for imaging and Quantity One (Bio-Rad version 4.6.2) was used for quantitative analysis. The gray value ratio of the target protein to the internal reference (GAPDH) was regarded as the relative protein expression. Experiments were repeated three times to obtain the mean values.

### Immunohistochemical staining

The paraffin-embedded tumor tissues prepared from in vivo experiments were sectioned to a thickness of 4 μm and mounted on polylysine-coated slides for immunohistochemistry assays to detect protein expression levels of EMT factors. The indirect streptavidin-peroxidase method kit (ZSGB-bio, Beijing, China) was used according to the protocol provided by the manufacturer. Briefly, the sections were deparaffinized in xylene and rehydrated in ethanol of gradient concentrations. Antigen retrieval was performed by heating at 100 °C in 10 mM citrate buffer (Cwbio, Beijing, China) in a pressure cooker for 20 min. The sections were treated with 3% H_2_O_2_ for 25 min to quench endogenous peroxidase activity, and with sheep serum for 30 min to block the non-specific binding. Then, the sections were incubated in a humidity chamber with the following antibodies overnight at 4 °C: anti-E-cadherin (Cat. No. 20874–1-AP, 1:100, PTG, USA), anti-Vimentin (Cat. No. 22031–1-AP, 1:100, PTG, USA). The biotinylated secondary antibody, horseradish peroxidase streptavidin (Cat. No. ab205718, 1:4000, Abcam, USA), and diaminobenzidine (Cat. No. G1211, Servicebio, China) were used successively as the detection reagents. Finally, the sections were counterstained with hematoxylin (Cat. No. G1004, Servicebio, China) for 1 min. Negative controls without primary antibody were used to exclude nonspecific binding.

### Statistical analysis

All data are shown as the mean ± SEM. Graphpad Prism 6.0 (GraphPad, Inc., USA) was used for statistical analysis. The statistical analysis methods included Student’s t-test and Pearson’s correlation analysis.

## Results

### PinX1 is downregulated and EMT is promoted in nasopharyngeal CD133^+^ CSCs

**Fig. 1:**
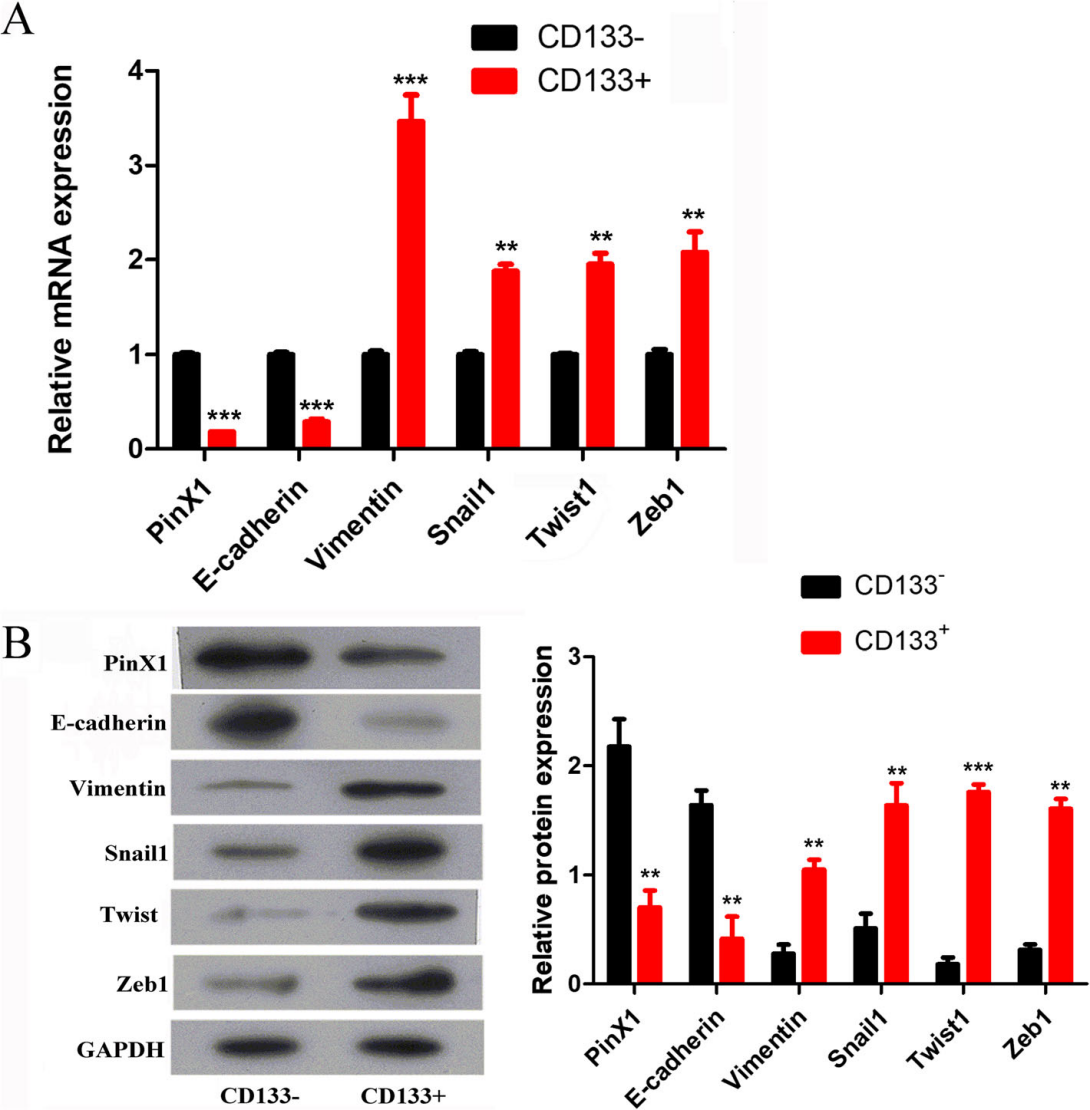
PinX1 is down-regulated and EMT process is promoted in nasopharyngeal CD133^+^ CSCs. **a** The mRNA expressions of PinX1, *E-cadherin*, *Vimentin*, *Snail1*, *Twist1*, and *Zeb1* in nasopharyngeal CD133^+^ CSCs and CD133^−^ cells as determined by RT-qPCR. **b** The protein levels of PinX1, E-cadherin, Vimentin, Snail1, Twist1, and Zeb1 in nasopharyngeal CD133^+^ CSCs and CD133^−^ cells as measured by western blot analysis. The grey value of the protein was normalized to that of the corresponding GAPDH. ** *p* < 0.01 vs. nasopharyngeal CD133^−^ CSCs, *** *p* < 0.001 vs. nasopharyngeal CD133^−^ CSCs

The expression of PinX1, E-cadherin, Vimentin, Snail1, Twist1, and Zeb1 in nasopharyngeal CD133^+^ CSCs and CD133^−^ cells was determined by qRT-PCR and western blot analysis. The mRNA levels of *PinX1* and *E-cadherin* were found to be decreased in nasopharyngeal CD133^+^ CSCs in comparison to those in CD133^−^ cells, but the mRNA levels of *Vimentin*, *Snail1*, *Twist1*, and *Zeb1* were elevated (all *p* < 0.05; Fig. 1a). When compared to those in CD133^−^ nasopharyngeal CSCs, the CD133^+^ CSCs showed a decrease in the protein levels of PinX1 and E-cadherin and an increase in the protein levels of Vimentin, Snail1, Twist1, and Zeb1 (all *p* < 0.05; Fig. 1b). These results proved that PinX1 was poorly expressed while EMT was highly promoted in nasopharyngeal CD133^+^ CSCs.

### Overexpression of PinX1 suppresses EMT by inhibiting EMT-related transcription factors in nasopharyngeal CD133^+^ CSCs

**Fig. 2:**
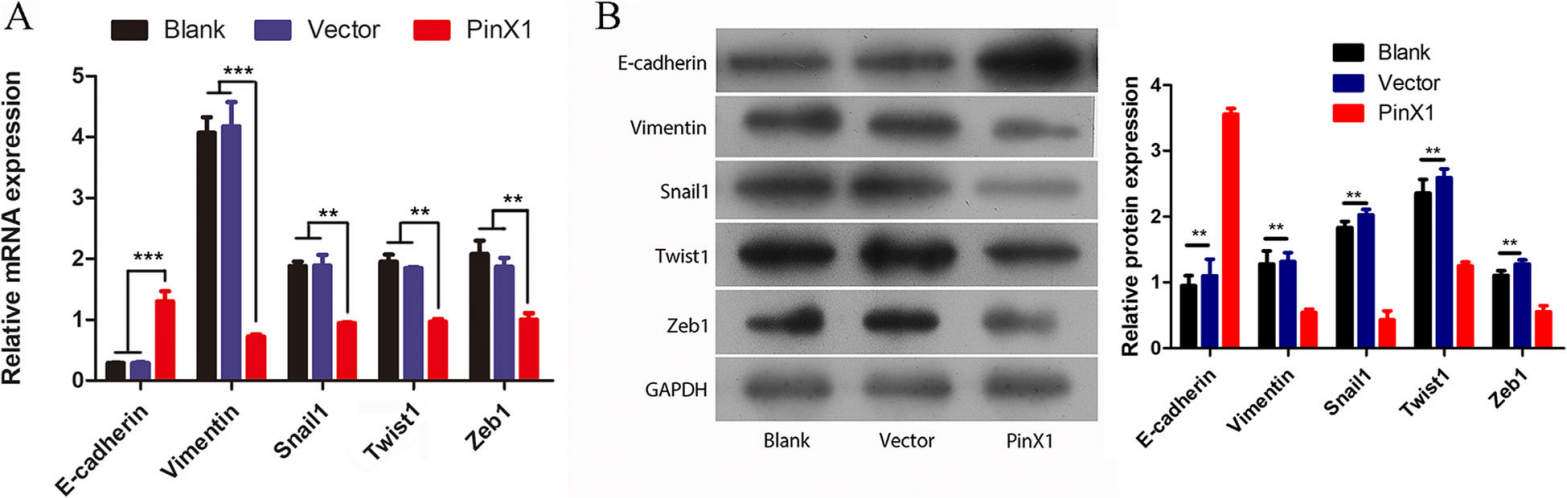
Overexpression of PinX1 suppresses cell EMT process in nasopharyngeal CD133^+^ CSCs. **a** The mRNA expressions of *E-cadherin*, *Vimentin*, *Snail1*, *Twist1*, and *Zeb1* in nasopharyngeal CD133^+^ CSCs and cells transfected with empty vector and pcDNA3.0-PinX1 as determined by RT-qPCR. **b** The protein levels of E-cadherin, Vimentin, Snail1, Twist1, and Zeb1 in nasopharyngeal CD133^+^ CSCs and cells transfected with empty vector and pcDNA3.0-PinX1 as determined by Western blot. The grey value of the protein was normalized to that of the corresponding GAPDH. ** p < 0.01 vs. control, *** p < 0.001 vs. control

We first examined whether PinX1 could regulate EMT in nasopharyngeal CD133^+^ CSCs by transducing them with pcDNA3.0-PinX1. The epithelial marker E-cadherin and the mesenchymal marker Vimentin, related to the EMT process, were detected using RT-qPCR and western blot analyses. E-cadherin was significantly upregulated with PinX1 overexpression, whereas Vimentin showed the opposite pattern, as compared to levels in the blank and empty vector groups (all *p* < 0.05; Fig. 2a). Similar results were found based on western blot assays (all p < 0.05; Fig. 2b). These results imply that the overexpression of PinX1 might suppress EMT in nasopharyngeal CD133^+^ CSCs. In our previous work [13], the overexpression of PinX1 was demonstrated to significantly inhibit proliferation, migration, and invasion of these cells, and therefore, PinX1 might inhibit NPC metastasis by suppressing EMT in nasopharyngeal CD133^+^ CSCs.

To reveal the potential mechanism through which PinX1 inhibits NPC aggressiveness, the relative mRNA and protein levels of a series of EMT-related transcription factors including Snail1, Twist1, and Zeb1 were further analyzed by RT-qPCR and western blot assays. In nasopharyngeal CD133^+^ CSCs with stable PinX1 overexpression, the EMT-inducing transcription factors Snail1, Twist1, and Zeb1 were significantly downregulated as compared to levels in the blank and empty vector groups (all *p* < 0.05; Fig. 2a). Similar results were found based on western blot assays (all p < 0.05; Fig. 2b). Snail1, Twist1, and Zeb1 can efficiently inhibit the cell–cell adhesion molecule E-cadherin, and this suppression is considered a hallmark of activated EMT. Thus, PinX1 might inhibit Snail1, Twist1, and Zeb1 expression, leading to the deregulation of E-cadherin, and inhibiting nasopharyngeal CD133^+^ CSC EMT. These results demonstrated that PinX1 is involved in EMT and that its overexpression can inhibit this process in nasopharyngeal CD133^+^ CSCs.

### Overexpression of P53 partially promotes the effects of PinX1 overexpression on EMT, migration, and invasion in nasopharyngeal CD133^+^ CSCs

**Fig. 3:**
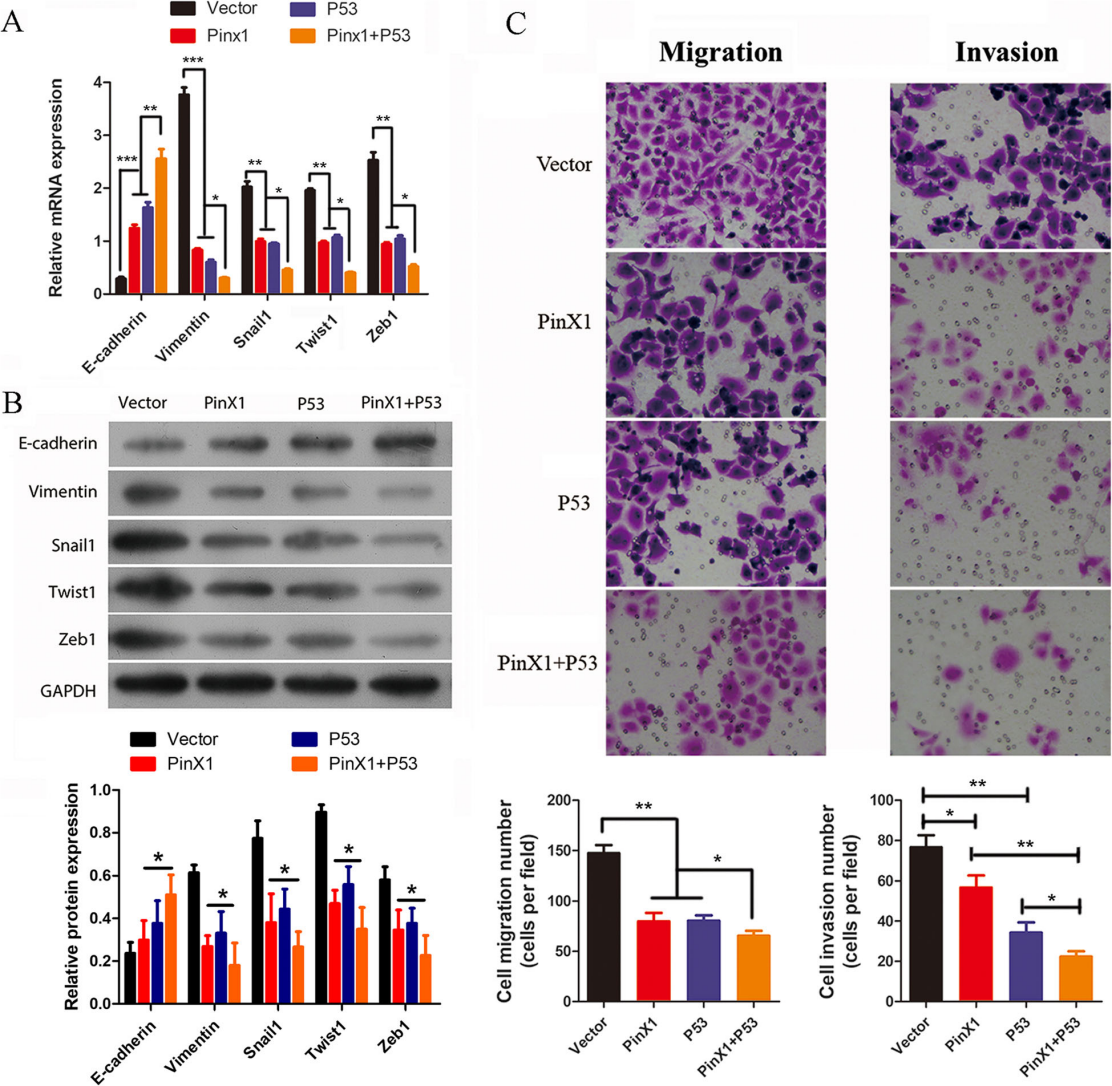
Overexpression of P53 partially promoted the effects of PinX1overexpreesion on cell EMT, migration and invasion in nasopharyngeal CD133^+^ CSCs. **a** The mRNA expressions of *E-cadherin*, *Vimentin*, *Snail1*, *Twist1*, and *Zeb1* in nasopharyngeal CD133^+^ CSCs transfected with empty vector and pcDNA3.0-PinX1, pcDNA3.0-P53 and pcDNA3.0-PinX1 + pcDNA3.0-P53 as determined by RT-qPCR. **b** The protein levels of E-cadherin, Vimentin, Snail1, Twist1, and Zeb1 in nasopharyngeal CD133^+^ CSCs transfected with empty vector and pcDNA3.0-PinX1, pcDNA3.0-P53 and pcDNA3.0-PinX1 + pcDNA3.0-P53as determined by Western blot. The grey value of the protein was normalized to that of the corresponding GAPDH. **c** Cell migration and invasion following transfection as measured by Transwell assay. * *p* < 0.05 vs. control, ** *p* < 0.01 vs. control, *** p < 0.001 vs. control

To further investigate the mechanisms underlying the involvement of PinX1 in nasopharyngeal CD133^+^ CSC EMT, we next determined whether P53 expression could affect this process and cell invasion. RT-qPCR and western blot assays showed that the overexpression of P53 downregulated Vimentin expression and upregulated E-cadherin expression similar to that observed with PinX1 overexpression. The expression of EMT-related transcription factors including Snail1, Twist1, and Zeb1 was also significantly repressed with P53 overexpression compared to that in the empty vector group. In addition, pcDNA3.0-PinX1 and pcDNA3.0-P53 co-transfection further inhibited Vimentin, Snail1, Twist1, and Zeb1 expression and promoted E-cadherin expression (Fig. 3a, b). Moreover, the overexpression of P53 significantly inhibited cell migration/invasion ability, and the co-overexpression of P53 and PinX1 further suppressed cell migration/invasion ability (Fig. 3c). Our previous work demonstrated that the overexpression of PinX1 upregulates P53 expression. Thus, PinX1 might upregulate P53 expression in nasopharyngeal CD133^+^ CSCs to synergistically inhibit EMT.

### The inhibition of miR-200b blocks the effects of PinX1 overexpression on EMT, migration, and invasion in nasopharyngeal CD133^+^ CSCs

**Fig. 4:**
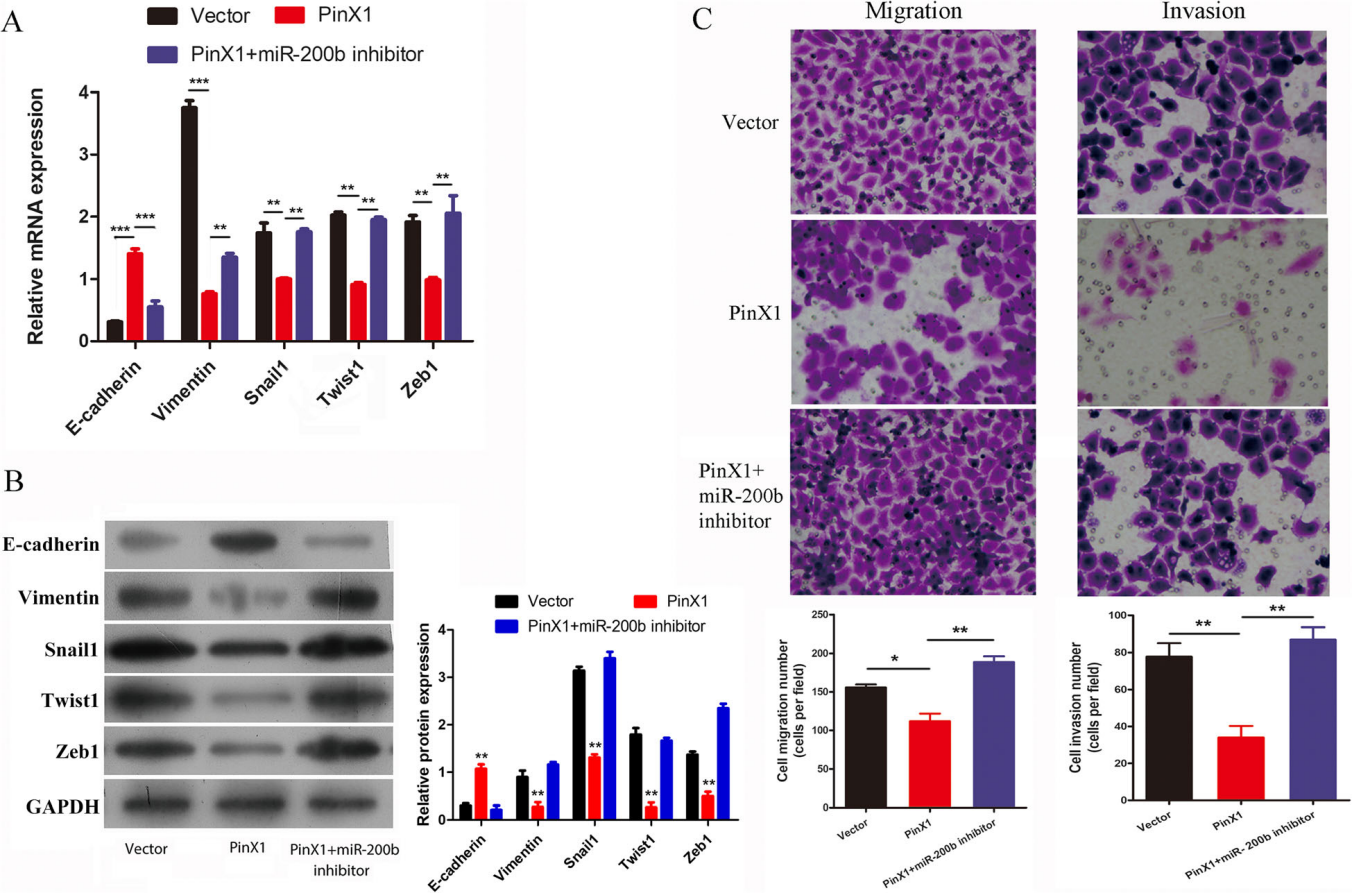
Inhibition of miR-200b blocked the effects of PinX1overexpreesion on cell EMT, migration and invasion in nasopharyngeal CD133^+^ CSCs. **a** The mRNA expressions of *E-cadherin*, *Vimentin*, *Snail1*, *Twist1*, and *Zeb1* in nasopharyngeal CD133^+^ CSCs transfected with empty vector and pcDNA3.0-PinX1 and pcDNA3.0-PinX1 + miR-200b inhibitor as determined by RT-qPCR. **b** The protein levels of E-cadherin, Vimentin, Snail1, Twist1, and Zeb1 in nasopharyngeal CD133^+^ CSCs transfected with empty vector and pcDNA3.0-PinX1 and pcDNA3.0-PinX1+ miR-200b inhibitor as determined by Western blot. The grey value of the protein was normalized to that of the corresponding GAPDH. **c** Cell migration and invasion following transfection as measured by Transwell assay. * p < 0.05 vs. control, ** p < 0.01 vs. control, *** p < 0.001 vs. control

Further, we investigated whether miR-200b plays an important role in EMT and nasopharyngeal CD133^+^ CSC invasion ability. RT-qPCR and western blot analysis showed that the overexpression of PinX1 significantly downregulated Vimentin, Snail1, Twist1, and Zeb1 expression and upregulated E-cadherin expression; however, the co-transfection of pcDNA3.0-PinX1 and a miR-200b inhibitor diminished the effects of PinX1 in nasopharyngeal CD133^+^ CSCs (Fig. 4a, b). Moreover, PinX1 overexpression inhibited cell migration/invasion ability; however, pcDNA3.0-PinX1 and miR-200b inhibitor co-transfection prevented these effects in nasopharyngeal CD133^+^ CSCs (Fig. 4c). Thus, these results confirmed that PinX1 suppresses EMT via a miR-200b-dependent pathway.

### Overexpression of P53 promotes nasopharyngeal CD133^+^ CSC proliferation and sphere formation ability in vitro but miR-200b inhibition blocks the effects of PinX1 overexpression

**Fig. 5:**
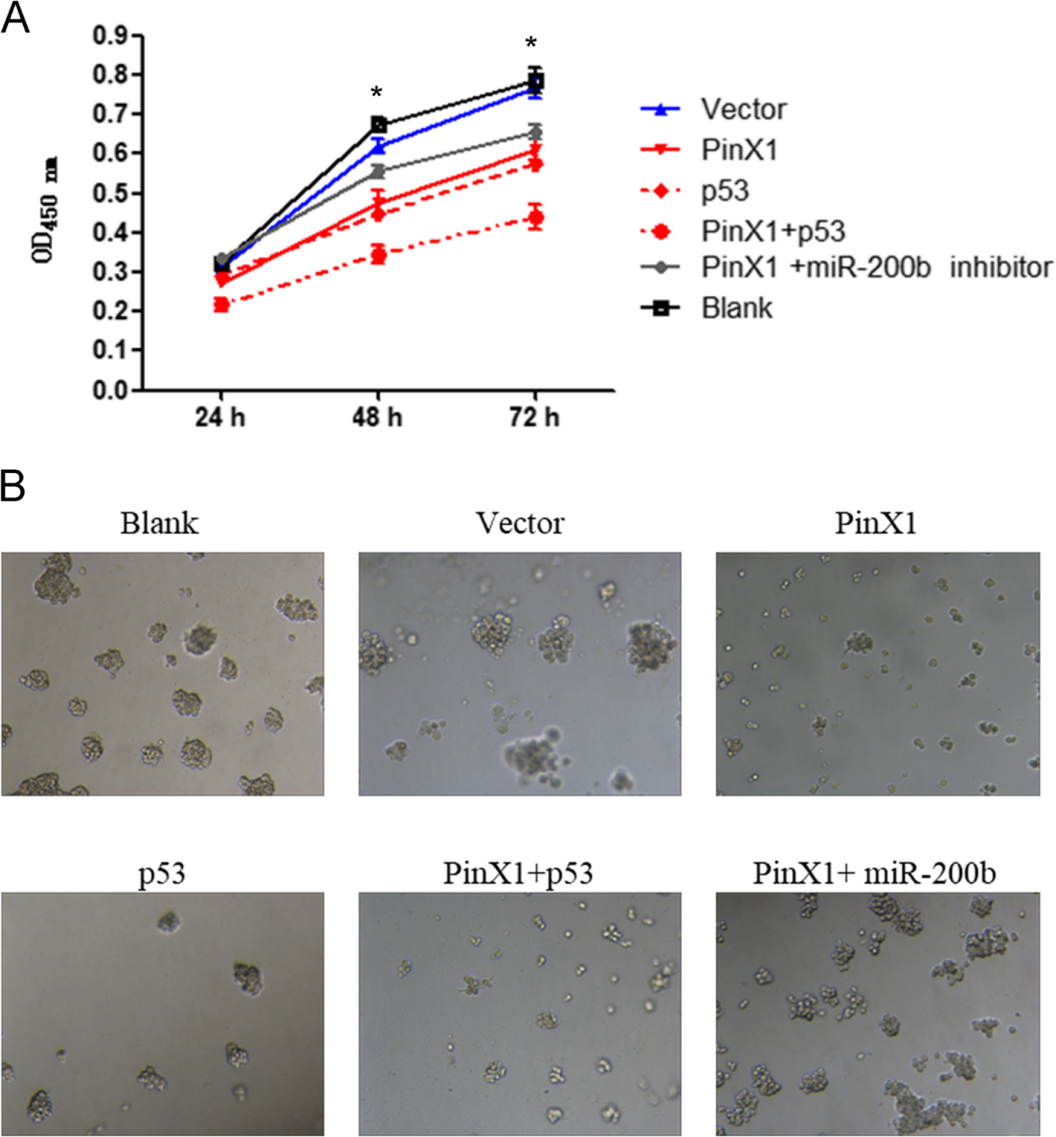
Overexpression of P53 promoted while inhibition of miR-200b blocked the effects of PinX1 overexpression on cell proliferation and sphere formation ability of nasopharyngeal CD133^+^ CSCs in vitro. **a** The cell viability curves of nasopharyngeal CD133^+^ CSCs following transfection as measured by CCK-8 assay. **b** Sphere formation ability of nasopharyngeal CD133^+^ CSCs following transfection in vitro as determined by colony formation analysis. * p < 0.05 vs. control

Finally, the cell proliferation and in vitro sphere formation ability of nasopharyngeal CD133^+^ CSCs following transfection were determined via CCK8 and colony formation analyses. The relative viability of nasopharyngeal CD133^+^ CSCs following transfection in each group is shown in Fig. 5a. No differences at the 24-h time point were found among the groups (*p* > 0.05). In contrast to that observed in the blank and vector groups, the PinX1, P53, and PinX1 + P53 groups exhibited inhibited cell viability at 48 and 72 h (all *p* < 0.05). However, this did not differ significantly among the blank, vector, and PinX1 + miR-200b inhibitor groups (p > 0.05). To further elucidate the effects of PinX1 on tumor growth, colony formation analyses were performed as shown in Fig. 5b. Compared to those in the blank and vector groups, stem cell spheres of different sizes and irregular shapes were obviously reduced in PinX1, P53, and PinX1 + P53 groups, while no significant differences were observed in the PinX1 + miR-200b inhibitor group. Altogether, the overexpression of PinX1 inhibits nasopharyngeal CD133^+^ cancer stem cell proliferation by activating the miR-200b and P53 pathway.

### The inhibition of miR-200b blocks the effects of PinX1 overexpression on nasopharyngeal CD133^+^ CSCs tumorigenesis and EMT in vivo

**Fig. 6:**
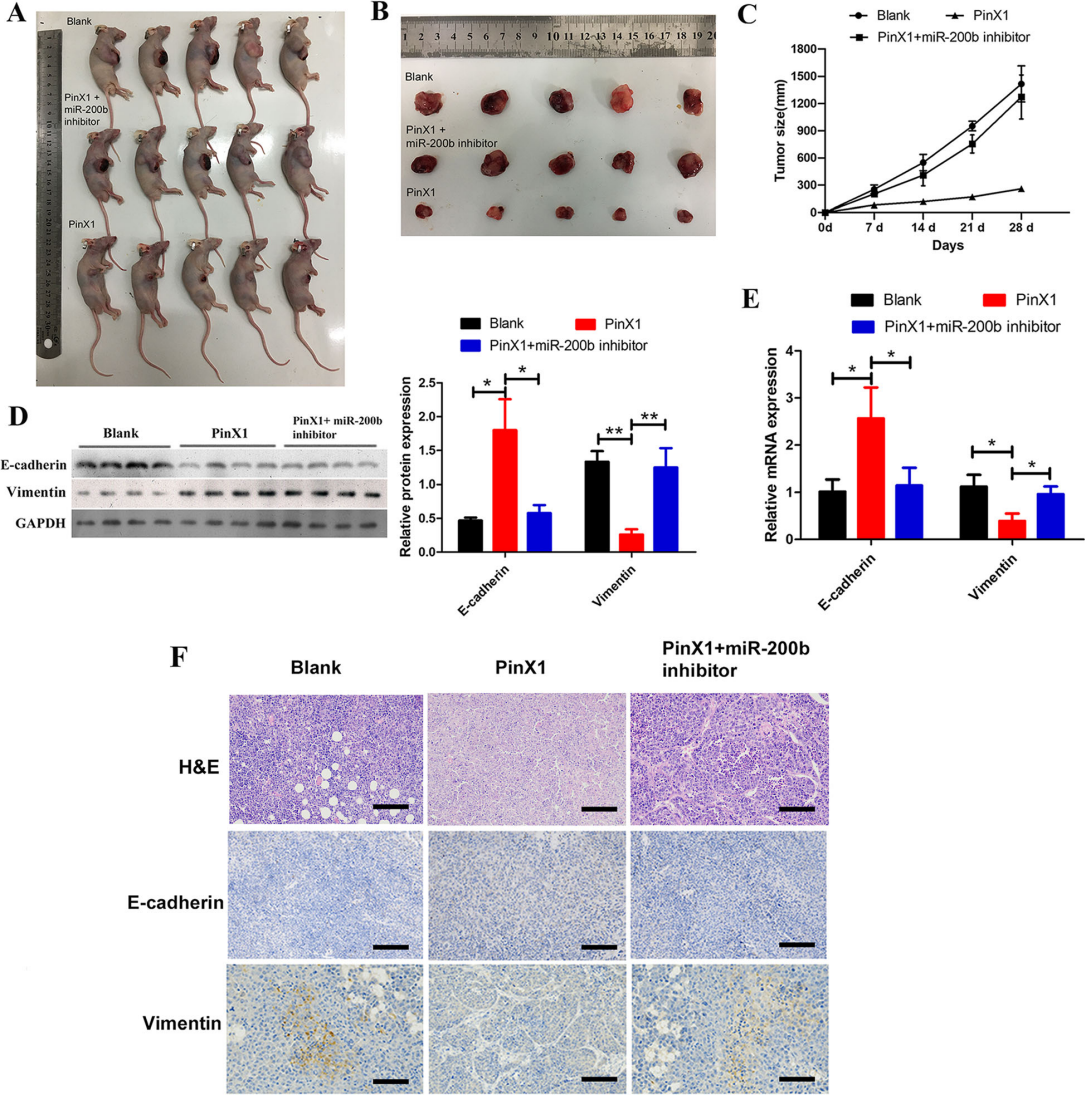
Inhibition of miR-200b blocked the effects of PinX1 overexpression on cell proliferation and EMT of nasopharyngeal CD133^+^ CSCs in vivo. **a** The xenograft mice models bearing tumours originating from CD133^+^ CSCs, *n* = 5 /group. **b** tumour volume was periodically measured for each mice. **c** tumour growth curves was plotted. **d** The protein level of Key EMT-related markers was detected by western blot in xenografts derived from nasopharyngeal CD133^+^ CSCs after overexpression of PinX1 and co-inhibition of miR-200b. The grey value of the protein was normalized to that of the corresponding GAPDH. **e** The mRNA level of Key EMT-related markers was detected by RT-qPCR in xenografts derived from nasopharyngeal CD133^+^ CSCs after overexpression of PinX1 and co-inhibition of miR-200b. **f** Representative H&E staining of primary cancer tissues are shown as well as immunohistochemistry (IHC) detection of E-cadherin and Vimentin in xenografts derived from nasopharyngeal CD133^+^ CSCs. Magnification × 400. Scale bar: 30 μm. * p < 0.05 vs. control, ** p < 0.01 vs. control

Furthermore, an in vivo tumour formation experiment by subcutaneously injecting CD133^+^ CSCs without any transfection (Blank group), CD133^+^ CSCs transfected with pcDNA3.0-PinX1 (PinX1 group) and CD133^+^ CSCs transfected with pcDNA3.0-PinX1 and miR-200b inhibitor (PinX1 + miR-200b inhibitor group) into nude mice were conducted to confirm the effects concerning PinX1 and miR-200b axis (Fig. 6). After 28 days of implantation, the mice injected with PinX1 overexpression cells had smaller tumour burdens than blank group while no significant differences were observed in the PinX1 + miR-200b inhibitor group (Fig. 6a, b). And the growth rate of tumor on mice injected with PinX1 overexpression cells was significantly lower than blank group while no significant differences were observed in the PinX1 + miR-200b inhibitor group (Fig. 6c). In accord with in vitro results, overexpression of PinX1 strongly suppressed the protein and mRNA expression levels of epithelial marker E-cadherin and promoted the expression of mesenchymal marker Vimentin that related to the EMT process in vivo, while the effects could be reversed by co-inhibition of miR-200b (Fig. 6d and e). These tumours also exhibited an increase in E-cadherin expression and a reduction in Vimentin expression in xenografts derived from PinX1 overexpression nasopharyngeal CD133^+^ CSCs, while the expression levels were reversed by co-inhibition of miR-200b via immunohistochemistry (Fig. 6f). These results showed PinX1 overexpression significantly inhibits tumorigenesis and EMT in vivo while miR-200b inhibition blocks the effects, indicating that miR-200b signal pathway played a vital role on PinX1 regulating aggressive behaviors of nasopharyngeal CD133^+^ CSCs.

## Discussion

Although multimodal treatment for the local control of NPC has achieved great advances, with an improved 5-year survival rate (approximately 80%), local recurrence and distant metastasis are still primarily responsible for treatment failure and death associated with NPC [20]. Therefore, a better understanding of the underlying mechanism of NPC metastasis is the key to explore novel treatment strategies for patients with this disease. CSCs, a group of “tumor-initiating cells” possessing the capacity to initiate tumor growth, self-renewal properties, and multi-drug resistance, have been found to be highly related to tumor occurrence, development, and metastasis [21]. Over the years, increasing studies have focused on understanding the biological properties and mechanisms of CSC formation to develop novel strategies to identify these stem-like cancer cells and to target them specifically [22–24]. EMT is considered an important process that leads to tumor invasion and distant metastasis, and it has become an important biological characteristic of CSCs that confers new biological behaviors such as chemotherapy resistance, anti-radiation properties, recurrence, and distant metastasis [25]. Guen et al. [26] demonstrated that EMT programs promote basal mammary stem cell and tumor-initiating cell stemness by inducing primary ciliogenesis and Hedgehog signaling. Further, Nomura et al. [27] found that the overexpression of CD133 can increase the expression and secretion of IL1 beta (IL1B), which activates an autocrine signaling loop that upregulates NF-kappa B signaling, EMT, and cellular invasion. These results together indicate that EMT and CSCs are mutually interdependent and together confer specific biological behaviors to the tumor.

In this study, we demonstrated that EMT participates in NPC progression. The epithelial marker E-cadherin was decreased, and the mesenchymal marker Vimentin was increased in nasopharyngeal CD133^+^ CSCs compared to levels in nasopharyngeal CD133^−^ CSCs. One important characteristic of EMT is the decrease in cadherin expression and the increase in vimentin expression, which indicates that EMT was significantly promoted in nasopharyngeal CD133^+^ CSCs. To provide a more comprehensive understanding of tumor EMT, a series of EMT-related transcription factors including Snail1, Twist1, and Zeb1 were detected. Twist1 is the most important regulator of EMT and is significantly associated with expression of the mesenchymal markers fibronectin and vimentin [28]. Zhu et al. [29] found that Twist11 is involved in EMT in esophageal cancer and cancer-associated fibroblasts and plays a vital role in tumor growth in vivo. Another transcription factor that is closely related to EMT is Snail1, a zinc finger transcriptional repressor that can induce morphological and molecular changes that are characteristic of EMT in breast cancer cells [30]. In addition, Ota et al. [31] found that Snail-induced EMT maintains the CSC-like phenotype, and enhances sphere formation capability, chemoresistance, and invasive ability in head and neck squamous cell carcinoma cells. Moreover, Zeb1, a powerful EMT-related transcription factor, significantly mediates doxorubicin resistance and mesenchymal characteristics in hepatocarcinoma cells [32]. Therefore, understanding the function of transcriptional factors such as Snail1, Twist1, and Zeb1 is necessary to achieve a more comprehensive understanding of tumor EMT. The high expression of Snail1, Twist1, and Zeb1 in nasopharyngeal CD133^+^ CSCs indicated that EMT was promoted via the upregulation of these markers.

Telomerase and its core component human telomerase reverse transcriptase (hTERT) are crucial players in cancer metastasis and stemness. El-Badawy et al. [33] investigated the function of hTERT in EMT in CSCs and suggested that targeting this marker might improve the elimination of breast CSCs by coordinating with EMT through a feedback loop. Using gastric cancer as a model, Liu et al. [34] also demonstrated that hTERT stimulates EMT and induces cancer cell stemness, thereby promoting cancer metastasis and recurrence. Therefore, targeting hTERT might prevent cancer progression by inhibiting EMT and CSCs. Our previous work demonstrated that the overexpression of PinX1 significantly decreases hTERT expression, inhibits proliferation, migration, and invasion, and induces apoptosis in nasopharyngeal CD133^+^ CSCs by regulating TRFs and Mad1/c-Myc/P53 pathways [13]. However, the ability of PinX1 to alter the biology of cancer cells needed to be further clarified. Specifically, we found that EMT is significantly inhibited by PinX1 overexpression. Further, in this study, E-cadherin was found to be upregulated and Vimentin, Snail1, Twist1, and Zeb1 were downregulated when PinX1 expression was promoted. Hence, the inhibitory effect on EMT was suggested to be due to PinX1-mediated suppression of the expression of key transcriptional factors involved in this process. In addition, the overexpression of PinX1 was found to diminish the migration and invasion of nasopharyngeal CD133^+^ CSCs. These results confirmed the role of PinX1 in suppressing tumor aggressiveness in NPC cell lines.

To further reveal the mechanisms underlying the involvement of PinX1 in EMT in nasopharyngeal CD133^+^ CSCs, the probable related pathways were determined. Since PinX1 was previously found to be associated with P53 and MYC pathways [13], this study further clarified whether P53 is involved in modulating these pathways and suppressing NPC aggressiveness. The expression of E-cadherin was found to be upregulated while the expression of Vimentin and EMT-related transcription factors including Snail1, Twist1, and Zeb1, was significantly suppressed upon P53 overexpression. Co-transfection with pcDNA3.0-PinX1 and pcDNA3.0-P53 further inhibited Vimentin, Snail1, Twist1, and Zeb1 expression and promoted E-cadherin expression. In addition, the overexpression of PinX1 significantly upregulates the expression of P53 in nasopharyngeal CD133^+^ CSCs [13]. These results confirmed that P53 plays a crucial role in PinX1-regulated EMT and tumor aggressiveness in NPC.

Furthermore, this study investigated the function of miR-200b in PinX1-regulated EMT in nasopharyngeal CD133^+^ CSCs. MicroRNAs (miRNAs) are an important class of tumor suppressors or oncogenes that function by regulating their target genes through mRNA degradation, post-transcriptional repression, or promoter activation [35, 36]. These molecules have been recently reported to be closely associated with tumor growth, metastasis, and angiogenesis via transcription factor P53 pathways [37, 38]. Taewan Kim et al. [39] reported that P53 suppresses EMT by transactivating miR-200 family members and then repressing the expression of ZEB1 and ZEB2. Here, we found that the inhibition of miR-200b significantly blocks the effects of PinX1 overexpression on EMT, migration, and invasion in nasopharyngeal CD133^+^ CSCs. In addition, the proliferation and in vitro sphere formation abilities of nasopharyngeal CD133^+^ CSCs were significantly recovered after inhibiting miR-200b. These data together revealed that miR-200b is a key target of PinX1 during the inhibition of EMT.

P53-induced miRNAs played a key role in tumor proliferation, metastasis, and angiogenesis by regulating EMT during the initiation and development of cancer; for example, P53-induced miR-1249 might suppress colorectal cancer (CRC) growth, metastasis, and angiogenesis by targeting VEGFA and HMGA2, in addition to regulating the Akt/mTOR pathway and EMT processes [19]. In addition, Laudato et al. [18] also found that P53-induced miR-30e-5p might inhibit CRC invasion and metastasis by targeting ITGA6 and ITGB1. Here, we observed that the overexpression of PinX1 and P53 inhibits cell proliferation, migration, and invasion, but that the inhibition of miR-200b blocked these effects, in nasopharyngeal CD133^+^ CSCs. In addition, we found that PinX1 and P53 inhibit EMT by suppressing Snail1/Twist11/Zeb1 expression.

## Conclusions

In conclusion, we show here that PinX1 inhibits cell proliferation, migration, and invasion by regulating the P53/miR-200b-mediated transcriptional suppression of Snail1, Twist1 and Zeb1, consequently inhibiting EMT, in nasopharyngeal CD133^+^ CSCs. As these processes play a crucial role in the malignant progression of human NPC, our results have showed that P53/miR-200b axis may be a pivotal target for NPC therapy.

## Data Availability

All data generated or analysed during this study are included in this published article.

## Abbreviations

CCK8: Cell Counting Kit-8
CSCs: cancer stem cells
EMT: Epithelial–mesenchymal transition
FBS: Fetal bovine serum
GAPDH: Glyceraldehyde-3-phosphate dehydrogenase
hTERT: human telomerase reverse transcriptase
LGR5: Leucine-rich repeat-containing G protein-coupled receptor 5
miR-200b: MicroRNA-200b
miRNAs: MicroRNAs
NPC: Nasopharyngeal carcinoma
PinX1: Pin2/telomeric repeat factor 1-interacting telomerase inhibitor 1
RT-qPCR: Reverse transcription-quantitative polymerase chain reaction
